# CCR7 mediates the TNF-α-induced lymphatic metastasis of gallbladder cancer through the “ERK1/2 - AP-1” and “JNK - AP-1” pathways

**DOI:** 10.1186/s13046-016-0318-y

**Published:** 2016-03-24

**Authors:** HaiJie Hong, CaiLong He, SiYuan Zhu, YanHui Zhang, XiaoQian Wang, FeiFei She, YanLing Chen

**Affiliations:** Department of Hepatobiliary Surgery and Fujian Institute of Hepatobiliary Surgery, Fujian Medical University Union Hospital, 29 Xinquan Road, Fuzhou, 350001 China; Key Laboratory of Ministry of Education for Gastrointestinal Cancer, Fujian Medical University, 1 Xueyuan Road, Minhou, Fuzhou, 350108 China; Fujian Key Laboratory of Tumor Microbiology, Fujian Medical University, 1 Xueyuan Road, Minhou, Fuzhou, 350108 China

**Keywords:** Gallbladder cancer, CCR7, TNF-α, Cancer-related inflammation, Lymphatic metastasis

## Abstract

**Background:**

CC-chemokine receptor 7 (CCR7), which plays an important role in cell directional movement, is highly expressed in various cancers and positively related to lymph node metastasis. The inflammatory cytokine tumour necrosis factor (TNF)-α promotes tumour progression and lymph node metastasis in gallbladder cancer (GBC). However, the expression of CCR7 in GBC is unclear, and its role in the TNF-α-induced lymphatic metastasis of GBC requires further research.

**Methods:**

The expression of CCR7 in clinical samples was detected by immunohistochemistry, and the relationship between CCR7 and clinicopathological factors or the TNF-α level of the bile was analyzed. After treatment with various concentrations of TNF-α, CCR7 expression in GBC cell lines was measured by Western blotting. The relative luciferase reporter assay, site-directed mutagenesis and chromatin immunoprecipitation were used to analyze the promoter activity and transcriptional regulation of CCR7. MAPKs inhibitors were used to explore the upstream signalling molecules of AP-1. We established a NOZ cell line stably expressing lentiviral CCR7 shRNA that effectively silenced the expression of CCR7, and to determine the role of TNF-α - CCR7 axis in the migration of GBC cells to the lymphatic system by transwell assays and animal experiments.

**Results:**

CCR7 was highly expressed in GBC samples. Higher expression of CCR7 was associated with American Joint Committee on Cancer (AJCC) staging and lymph node metastasis. Moreover, we found that CCR7 expression in GBC tissue was positively correlated with the levels of TNF-α in the bile, and that TNF-α enhanced the promoter activity and protein expression of CCR7 through the “ERK1/2-AP-1” and “JNK-AP-1” pathways. Finally, we revealed that TNF-α could promote GBC cell migration to lymphatic endothelial cells or lymph nodes through upregulation of CCR7 in vitro and in vivo.

**Conclusions:**

Our study suggests that CCR7 is highly expressed in GBC, and mediates the TNF-α-induced lymphatic metastasis of GBC through the “TNF-α - ERK1/2 - AP-1 - CCR7” and “TNF-α - JNK - AP-1 - CCR7” pathways.

## Background

Gallbladder cancer (GBC) is a rare and highly lethal disease. It accounts for 80-95 % of biliary tract malignancies, representing the most common cancer of the biliary tree [1]. GBC is a highly malignant tumour and has an extremely poor prognosis [2]. It tends to metastasize to the lymph nodes in its early stages, which seriously affects the prognosis. Chronic inflammation induced by gallstones is a major risk factor for GBC according to epidemiological investigations [1, 3].

The hypothesis that inflammation causes cancer dates back to the 19th century [4]. Follow-up studies indicate that inflammation can also promote tumour progression [5, 6]. Cytokines, such as tumour necrosis factor alpha (TNF-α), secreted by inflammatory cells play important roles in cancer-related inflammation [7]. In our previous study, we demonstrated that TNF-α can promote lymphangiogenesis and lymph node metastasis of GBC [8]. However, the specific mechanism explaining how GBC cells migrate to the lymphatic vessels remains to be elucidated. Whether TNF-α can promote this migration also requires further study.

Chemokines, a group of small molecule proteins (8–11 kd) secreted by endothelial cells, fibroblasts and macrophages, are another important cytokine in cancer-related inflammation. Chemokine receptors, which combine with the corresponding chemokines, are G-protein-coupled receptors, including CXC, CC, CX3C and XC receptors [9, 10]. Previous studies demonstrated that chemokine receptors and ligands play important roles in the directional movement of many cell types, such as inflammatory cell recruitment [11] and lymphocyte homing [12]. In 2001, Anja Muller first proposed that the process of tumour cell migration to target organs, including lymphatic vessels and lymph nodes, may be similar to that of lymphocyte homing, and suggested that chemokines and their receptors (CXCR4, CCR7 and CCR10) have a critical role in determining the metastatic destination of tumour cells [13]. Subsequently, many studies have confirmed this conclusion.

Chemokine receptors are primarily expressed on the membrane of cancer cells and mediate cancer cell migration to target organs by combination with their corresponding ligands. CC-chemokine receptor 7 (CCR7) is the specific receptor for chemokines CCL21/CCL19. It was demonstrated that CCR7 is associated with lymph node metastases of the malignancies [9, 10]. Research in various types of tumours, such as cervical cancer [14], oesophageal squamous cell carcinoma [15], gastric cancer [16], and melanoma [17], have confirmed that the CCR7-CCL21/CCL19 axis is involved in lymphatic metastases. However, the expression of CCR7 in GBC and its relationship with lymph node metastasis of GBC is unknown. Therefore, we hypothesized that TNF-α might promote lymphatic metastasis of gallbladder cancer via upregulation of CCR7.

In this study, we detected the expression of CCR7 in gallbladder cancer for the first time, and revealed the relationship between CCR7 and the clinicopathological factors of GBC patients. We found that CCR7 expression in GBC tissue is positively correlated with the levels of TNF-α in the bile, and further determined that TNF-α enhances the promoter activity and protein expression of the CCR7 gene through the “ERK1/2-AP-1 and JNK-AP-1” pathways. Moreover, we indicated that TNF-α could promote GBC cell migration to LECs in vitro and promote lymph node metastasis in the orthotopic transplantation of GBC through the upregulation of CCR7.

## Methods

### Specimens and immunohistochemistry

The study with clinical samples was approved by the Ethics Committee of the Medical Faculty of Fujian Medical University in China. Forty-two paraffin-embedded gallbladder cancer tissues and 22 para-carcinoma tissues were randomly obtained from the archives of the Department of Pathology at Fujian Medical University Union Hospital (from 2006 to 2013). Written informed consent was obtained from all patients before surgery. All patients included in this study had not been given any preoperative chemotherapy or other therapy such as radiotherapy. Immunohistochemistry analysis was performed as described previously [18]. Ten different fields were selected randomly from each slice and the percentage of positive cells was counted using a bright field light microscope (400×). The degree of immunostaining was reviewed and scored independently by two pathologists based on the intensity of staining: 0 (no staining), 1 (weak staining, light yellow), 2 (moderate staining, yellow brown), and 3 (strong staining, brown). The number of positive cells was graded as: 0 (<10 %), 1 (11–25 %), 2 (26–50 %), 3 (51–75 %) and 4 (>75 %). The two grades were multiplied together and the staining of each sample was evaluated as follows: negative, <1 points (-); positive, >1 point: 1 to 4 points (+), 5 to 8 points (++); 9 to 12 points (+++ ~ ++++).

### Cell culture and transfection

The human gallbladder cancer cell lines: NOZ (obtained from the Health Science Research Resources Bank in Japan), GBC-SD (purchased from the Shanghai Institutes for Biological Sciences in China) and SGC-996 (provided by the Tumour Cytology Research Unit, Medical College, Tongji University, Shanghai, China) were cultured in Dulbecco’s Modified Eagle’s Medium (DMEM, Gibco, USA) containing 10 % foetal bovine serum (FBS, Hyclone, USA). Human dermal lymphatic endothelial cells (HDLECs, purchased from Sciencell, USA) were incubated in endothelial cell medium (Sciencell). Cell transfection was carried out in 6-well plates at a density of 5 × 10^5^ cells/well or 24-well plates at a density of 1 × 10^5^ cells/well by using Lipofectamine 2000 (Invitrogen, USA) according to the manufacturer’s instructions. All of the cells were incubated at 37 °C in a humid atmosphere of 95 % air and 5 % CO2.

### Western blotting

Western blotting analysis was performed as described previously [19]. Monoclonal rabbit anti-human CCR7 antibody (1:2000), rabbit anti-human c-Jun (AP-1, 1:1000), rabbit anti-human p-c-Jun (p-AP-1, 1:1000), rabbit anti-human ERK1/2 (1:1000), rabbit anti-human p-ERK1/2 (1:500), rabbit anti-human JNK (1:1000), rabbit anti-human p-JNK (1:1000), rabbit anti-human p38MAPK (1:1000), and rabbit anti-human p-p38MAPK (1:1000) were purchased from Abcam. Monoclonal mouse anti-human β-actin (1:1000) was obtained from Santa Cruz. The signals were detected with an ECL kit (Beyotime, China) by following the manufacturer’s instructions.

### Real-time polymerase chain reaction

Real-time polymerase chain reaction (RT-PCR) was used to clone the CCR7 promoter fragment used in the site-directed mutagenesis and ChIP assay. Total RNA was isolated from the cultued cells using TRIzol reagent (Invitrogen) according to the manufacturer’s instructions. Total RNA was reverse transcribed using the Revert Aid First Strand cDNA Synthesis Kit (Thermo, USA) following the manufacturer’s instructions. These cDNA were used as the template for PCR amplification. The primers are shown in Table 1. The PCR conditions were as follows: 95 °C for 5 min, 95 °C for 30 s, and 61 °C for 30 s for 32 cycles.

**Table 1:** The primer sequences used in this study

Oligonucleotide*s*	Sequences (5′ → 3′)^a^	Length of products (bp)
CCR7 promoter cloning		
−1055/+74F	ccg *CTCGAG* CAGCCCAAACATCCAACT	1129
−725/+74F	ccg *CTCGAG* GTGATGGTTGCATAACTCTG	799
−462/+74F	ccg *CTCGAG* GGAAAGCCATTTGGGACT	536
−243/+74F	ccg *CTCGAG* GGATCCTATGACCAGCGAC	317
−108/+74F	ccg *CTCGAG* TGGCTTCTCCGACAACTT	182
−1055/+74R	ccc *AAGCTT* CAGGGTCAGTCTGGGTGT	
Site-directed mutagenesis		
AP1mut1F	CTGTCACCCTCTGGCACCTTTCTTCTG	
AP1mut1R	CAGAAGAAAGGTGCCAGAGGGTGACAG	
AP1mut2F	CCTGCATGAGTTCAGGAGGGCTGGG	
AP1mut2R	CCCAGCCCTCCTGAACTCATGCAGG	
PCR for ChIP		
AP1 ChIP-F	CACCTTTCTTCTGCCCCTTTATC	134
AP1 ChIP-R	TCAAGCCCCTTTTAAGTTGTCGG	

^a^Lowercase letters represent the protective-bases; italic letters represent the restriction enzyme cutting site; underlined letters represent the bases of mutation

### Construction of CCR7 promoter luciferase reporter plasmids and dual-luciferase reporter assay

Primers (Table 1) containing an XhoI or BglII (Thermo) restriction site were used to amplify a series of 5′-deletion CCR7 gene promoter fragments that were connected to the plasmid PGL4.10-Basic vector (Promega, USA) carrying a firefly luciferase reporter gene. These recombinants were named PGL4-1055 (−1055 to +74 nt, relative to the transcription start site “ATG”), PGL4-725 (−725 to +74 nt), PGL4-462 (−462 to +74 nt), PGL4-243 (−243 to +74 nt) and PGL4-108 (−108 to +74 nt). Forty-eight hours after transfection with promoter vector, cells were lysed and the intracellular luciferase activity of the lysates was measured using the Dual-Luciferase Reporter Assay System (Promega) according to the manufacturer’s instructions. The relative luciferase units were obtained by comparison to the luciferase activity of the pRL-TK plasmid (plasmid carrying a renilla luciferase report gene as an internal reference). Luminescence measurement was carried out on a luminometer (Orion II Microplate Luminometer, Berthold Detection Systems, Germany).

### Identification of putative transcription factor binding sites

The web sites TFbind (http://tfbind.hgc.jp/) and Promoter Scan (http://www-bimas.cit.nih.gov/molbio/proscan/) were used to search for potential transcription factor binding sites.

### Site-directed mutagenesis

Site-directed mutagenesis was performed by overlap extension PCR as previously described [20]. The primers targeting the two mutation sites of the AP-1 binding sites are shown in Table 1.

### Chromatin immunoprecipitation (ChIP) assay

The ChIP assay was performed according to the manufacturer’s instructions using the EZ-Magna ChIP kit (Merck Millipore, Germany). An antibody against AP-1 (c-Jun, phosphor S63, Abcam, England) and negative control normal rabbit IgG were used for immunoprecipitation. The primers for PCR are shown in Table 1.

### AP-1 (c-Jun) siRNA oligonucleotide treatment of cells

The AP-1 (c-Jun) siRNA interference sequence has been described previously [21] (named siAP-1). It and the non-targeting control (named siNC) were synthesized chemically by GenePharma Co., Ltd. (Suzhou, China). The transient transfection was performed according to the manufacturer’s instructions.

### Construction of a stable NOZ cell line with lentiviral CCR7 shRNA

We designed three siRNA sequences targeting the CCR7 gene (si1, si2, si3) and identified one of them, si3 (5′-GCGUCCUUCUCAUCAGCAA-3′), to effectively knock down CCR7 expression in NOZ cells. We used this sequence to construct a lentiviral vector expressing a CCR7 shRNA (named LV-shCCR7) and employed a non-targeting sequence lentiviral vector as control (named LV-shNC). Both vectors were constructed by GenePharma Co., Ltd. (Suzhou, China). The lentiviral vectors were used to infect NOZ cells with puromycin treatment for 2 weeks to screen for stably infected cells.

### Transwell assay

Cell migration and invasion were assayed using transwells (24-well format) with 8 μm polycarbonate membranes (Millipore, USA). The upper side of the membrane was covered with matrigel matrix (diluted 1:7) (BD Biosciences, USA) and air-dried for 1 h of incubation at 37 °C. The lower side of the membrane was covered with 5 μg fibronectin (BD Biosciences). Cells were resuspended in endothelial cell medium (ECM) after 8 h of serum-free starvation. Then, 200 ul ECM medium with 1 × 10^4^ NOZ cells was seeded to the upper chamber 500 ul ECM medium with or without 2 × 10^4^ HDLECs was added to the bottom chamber as attractants. After incubation for 48 h, the cells on the upper surface of the membrane were removed and the cells at the bottom of the filter were fixed with a stationary liquid of 95 % ethanol and 5 % acetic acid for 30 min and then stained with haematoxylin and eosin (H&E). The number of cells in each chamber was counted in nine random different fields (400×) using a bright field light microscope. Each experiment was repeated in triplicate.

### Animal experiments

Male 6-week old BALB/c nude mice were purchased from Shanghai SLAC Laboratory Animals Co. (Shanghai, China). All operations were according to institutional guidelines and were approved by the Ethics Committee of the Medical Faculty of Fujian Medical University. The orthotopic xenograft models were established following the method by Qiang Du [22]. Forty-eight mice were randomly divided into two groups (NOZ group and LV-shCCR7 group). Two weeks after inoculation, these two groups were each randomly divided into two group (twelve mice/group), and exogenous TNF-α (2 μg⁄kg) was injected into the peritoneal cavity of the two groups (one of the NOZ group and one of the LV-shCCR7 group) three times a week. The others (control group) were injected with normal saline. Five weeks after cell injection, the mice were euthanized by exposure to CO2, and the primary tumours were dissected and excised.

### Statistics

The results are presented as the mean ± SD from at least three independent experiments. Data analyses were performed with SPSS software (SPSS 13.0) by the analysis chi-square test (*χ*2 test), Fisher exact probability test, variance (ANOVA) and *t*-test. A two-sided *P-value* < 0.05 was considered statistically significant.

## Results

**Fig. 1:**
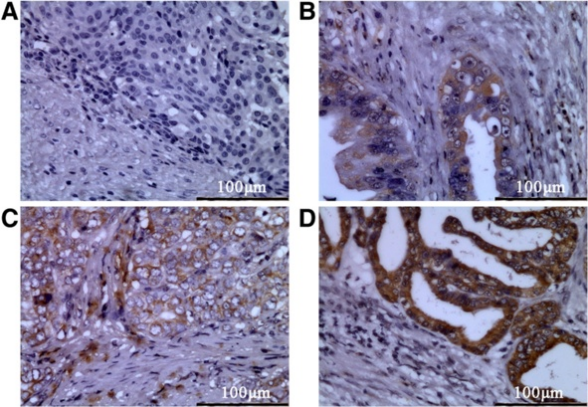
Representative IHC staining examples for CCR7 expression in gallbladder tissues (400×). **a** Negative, **b** weak, **c** moderate, **d** strong

1. **The expression of CCR7 in human gallbladder cancer and the relationship between CCR7 and clinicopathological factors.**
  We analyzed the expression of CCR7 in 42 clinical samples from gallbladder cancer patients and 22 normal gallbladder tissues adjacent to the carcinoma using immunohistochemistry. CCR7 was detected in the cytoplasm and membrane of GBC cells (Fig. 1b–d). However, negative or low levels of CCR7 expression were observed in the adjacent tissues. As shown in Table 2, CCR7 was expressed in 90.47 % (38/42) of GBC samples, which was significantly higher than that of adjacent tissues (22.73 %) (*P* < 0.05).

**Table 2:** The expression of CCR7 in the specimens of GBC and adjacent tissues

group	case number	CCR7 expression		positive rate (%)
		positive	negative	
GBC	42	38	4	90.47*
Adjacent-tissue	22	5	17	22.73

*P < 0.05
  The relationship between CCR7 expression and select clinicopathologic factors is shown in Table 3. Higher expression (+++ to ++++, strong staining) of CCR7 was associated with AJCC staging (I-IIa vs IIb-IV, *P* = 0.008 < 0.05) and lymph node metastasis (negative vs positive, *P* = 0.003 < 0.05), but not with age, gender, histological grade, and classification. Taken together, CCR7 overexpression may be involved in the lymph node metastasis of gallbladder cancer.

**Table 3:** The relationship between CCR7 and the clinicopathological factors of GBC

Factors	case number	CCR7		*χ* ^2^	P value
		- ~ ++	+++ ~ ++++		
Age(year)				0.475	0.491
<60	20	7	13		
≥60	22	10	12		
Gender				2.888	0.089
Male	19	5	14		
Female	23	12	11		
AJCC staging				6.959	0.008
I-IIa	17	11	6		
IIb-IV	25	6	19		
Histological grade				1.037	0.595
Poor	14	7	7		
Moderate	15	6	9		
High	13	4	9		
Classification				1.993	0.158
Adenocarcinoma	36	13	23		
Others	6	4	2		
Lymph node metastasis				8.968	0.003
Positive	24	5	19		
Negative	18	12	6
2. **CCR7 expression in GBC tissue is positively correlated with the levels of TNF-α in the bile.**
  Our previous study demonstrated that the level of TNF-α in the bile of GBC patients was significantly higher than that in patients with cholesterol gallbladder polyps [8]. To analyze the relationship between CCR7 expression and TNF-α levels in the bile, we detected the expression of CCR7 by immunohistochemistry in 20 specimens from the patients whose TNF-α levels in the bile had been detected by ELISA in our previous study [8]. As shown in Table 4, the level of TNF-α in the bile of GBC patients with strong staining of CCR7 was significantly higher than that of patients with negative/week/moderate staining (*P* < 0.05).

**Table 4:** The relationship between TNF-α levels in the bile and the expression of CCR7 in the tissues of GBC patients

CCR7 staining	case number	TNF-α(pg/ml)	P value
- ~ ++	4	416.6 ± 54.14	0.022
+++ ~ ++++	16	657.1 ± 45.49
3. **TNF-α promotes the expression of CCR7 in gallbladder cancer cell lines.**
  To explore whether TNF-α could promote the expression of CCR7, we first detected the protein levels of CCR7 in three GBC cell lines (NOZ, GBC-SD, and SGC-996), and measured the expression of CCR7 again in these cell lines after treatment with exogenous TNF-α. The expression of CCR7 protein in all three cell lines was confirmed. The levels of CCR7 protein in NOZ and SGC-996 cells were higher when compared to the GBC-SD cell lines (Fig. 2a, d). Thus, we employed the NOZ and SGC-996 cell lines in the next step of detection. GBC cells were incubated in 6-well plates and treated with various doses of TNF-α (5, 10, 20, and 50 ng⁄mL) for 12 h; the control groups were not treated with TNF-α. The expression of CCR7 was detected by Western blotting. As shown in Fig. 2b, e, TNF-α promoted the expression of CCR7 in NOZ and SGC-996 cell lines in a dose-dependent manner, and the peak effect appeared after treatment with 50 ng⁄mL (NOZ) and 20 ng/mL (SGC-996) TNF-α. Subsequently, we detected the CCR7 protein in NOZ and SGC-996 cells after treatment with 20 ng/mL TNF-α for 6, 12, and 24 h. The peak effect appeared after the 12-hour treatment with TNF-α (Fig. 2c, f). The NOZ cell line had the highest level of CCR7, so we used NOZ cells for further study.

**Fig. 2:**
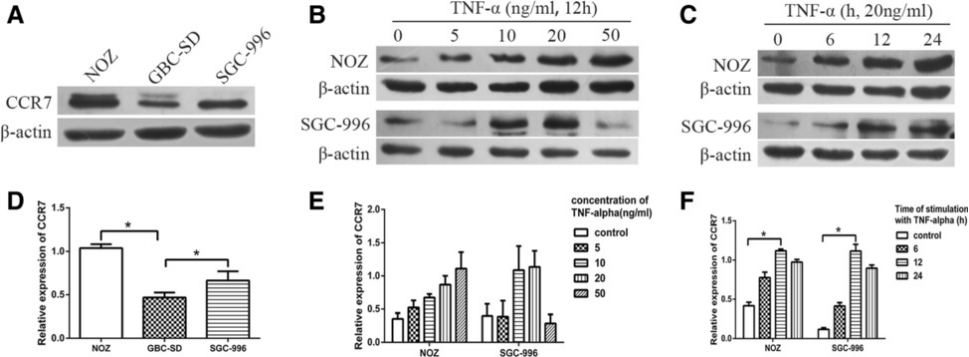
The expression of CCR7 in different GBC cell lines after treatment with TNF-α. **a**, **d** Expression of CCR7 in three human GBC cell lines (NOZ, GBC-SD, and SGC-996) detected by Western blotting. The levels of CCR7 protein in NOZ and SGC-996 cells were higher when compared to the GBC-SD cell lines. **b**, **e** GBC cells (NOZ and SGC-996) were treated with varying concentrations of TNF-α (5, 10, 20 and 50 ng⁄mL) for 12 h. The CCR7 protein level was measured by Western-blotting; the level increased in a dose-dependent manner. **c**, **f** CCR7 protein in NOZ and SGC-996 cells was detected after treatment with 20 ng/mL TNF-α for 6, 12, and 24 h. (*P < 0.05)
4. **TNF-α enhanced the promoter activity and protein expression of CCR7 through the “ERK1/2-AP-1 and JNK-AP-1” pathways.**
  To further explore the mechanism by which TNF-α upregulates CCR7, we first analyzed the promoter activity of CCR7 and determined the putative transcription factors binding to the promoter. A series of 5′-deletion constructs of the CCR7 promoter were transiently transfected into NOZ cells. As shown in Fig. 3a, the cells exhibited similar luciferase activities when transfected with either PGL4-1055/+74, PGL4-725/+74, PGL4-462/+74 or PGL4-243/+74, while luciferase activity markedly declined when transfected with PGL4-108/+74, suggesting that the region of -243 nt to -108 nt is crucial for the full activity of the CCR7 promoter. Two potential AP-1 binding sites were found (Fig. 3b) in the −243 nt to −108 nt region of the promoter by using the TFbind and Promoter Scan programs. The site-directed mutagenesis assay indicated that the AP-1(2)-binding site, but not the AP-1(1)-binding site, is crucial for the full activity of the CCR7 promoter (Fig. 3c). The ChIP assay was performed to confirm that the AP-1 transcription factor binds to the CCR7 promoter in vivo.

**Fig. 3:**
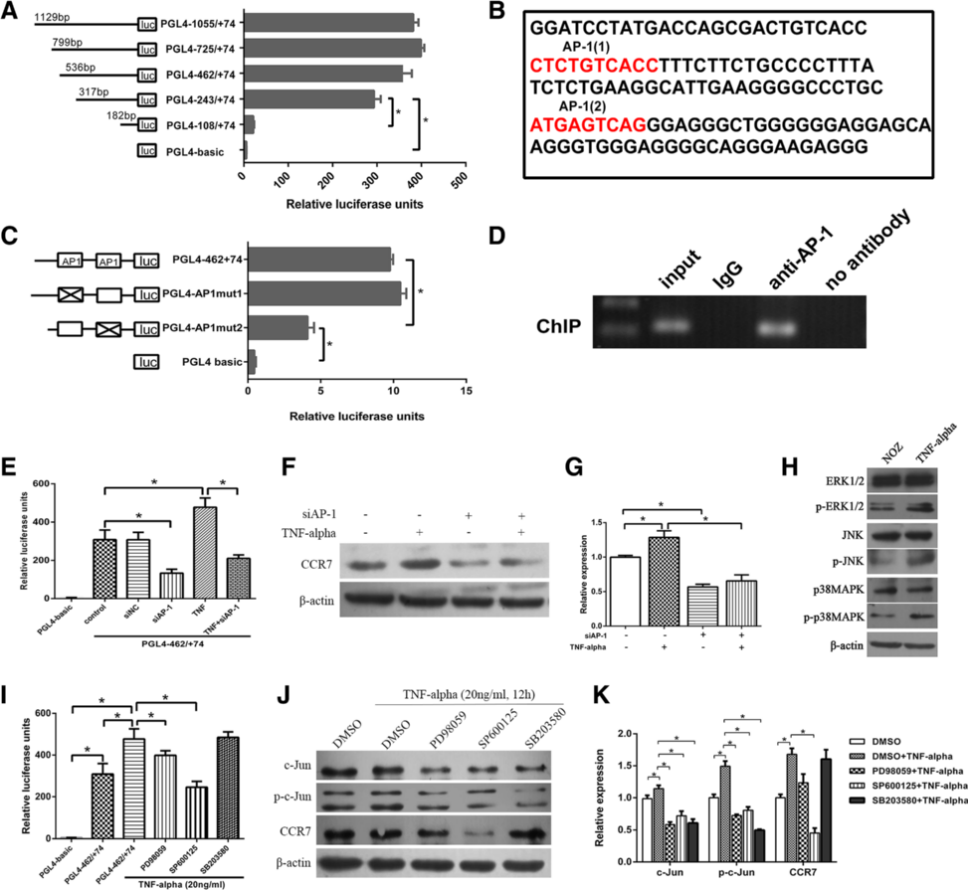
TNF-α enhanced the promoter activity and protein expression of the CCR7 gene through the “ERK1/2-AP-1 and JNK-AP-1” pathways. **a** Activity analysis of the CCR7 promoter. NOZ cells were transfected with 1 μg each of the CCR7 promoter constructs and 0.1 μg of pRL-TK. Each transfection was performed in duplicate. **b** Nucleotide sequence of the −243 to −108 region of the CCR7 promoter. The two putative AP-1 binding sites (CTCTGTCACC and ATGAGTCAG) were marked with red letters (named AP-1(1) and AP-1(2)). **c** Site-directed mutagenesis assay. The mutated construct PGL4-AP1mut2 but not PGL4-AP1mut1 exhibited lower activities than the non-mutated construct PGL4-462/+74. **d** ChIP assay. Chromatin from NOZ cells was immunoprecipitated with the anti-AP-1 antibody. The total extracted DNA (Input) and the immunoprecipitated samples were PCR-amplified using primers specific to the regions of the CCR7 promoter containing the AP-1(2) binding site (134 bp). A normal rabbit IgG and no antibody sample were also included as controls. **e**–**g** The effect of the TNF-α⁄AP-1 signalling pathway on the promoter activity and protein expression of the CCR7 gene. After transfection with AP-1 siRNA, the promoter activity and protein level of CCR7 were accordingly reduced, irrespective of treatment with TNF-α. **h** The expression of p-ERK1/2, p-JNK and p-p38MAPK was increased in NOZ cells after treatment with TNF-α. **i**-**k** The effect of inhibition of MAPK pathway members on the promoter activity and protein expression of CCR7. When treated with PD98059 (50 μM), SP600125 (10 μM) or SB203580 (20 μM), the expression of AP-1 and p-AP-1 in NOZ cells was reduced. However, the promoter activity and protein expression of CCR7 were significantly reduced only in the PD98059- and SP600125-treated groups (*P < 0.05). All of the experiments were repeated three times
  To determine the effect of the TNF-α⁄AP-1 signalling pathway on the promoter activity and protein expression of the CCR7 gene, we measured the luciferase intensity of the PGL4-462/+74 plasmid and CCR7 expression in NOZ cells treated with TNF-α or transfected with AP-1 (c-Jun) siRNA against AP-1 (siAP-1). As shown in Fig. 3e, f, g, the promoter activity and protein levels of the CCR7 gene were significantly reduced after transfection with siAP-1. TNF-α enhanced the expression of CCR7 and increased the luciferase activity of the CCR7 promoter. In contrast, when NOZ cells were transfected with siAP-1, the ability of TNF-α to upregulate luciferase activity and the protein expression of CCR7 were blunted.
  To determine which members of the MAPKs family (ERK1/2, JNK or p38MAPK) are involved in the upregulation of CCR7 induced by TNF-α, Western blotting was used to detect the expression of ERK1/2, p-ERK1/2, JNK, p-JNK, p38MAPK and p-p38MAPK in NOZ cells with or without treatment with TNF-α. The expression of p-ERK1/2, p-JNK and p-p38MAPK was increased (Fig. 3h). Thus, we assessed the effects of MAPK pathway inhibitors on the luciferase activity of the CCR7 promoter and the protein expression of AP-1, p-AP-1 and CCR7. As shown in Fig. 3i, j, k, treatment of NOZ cells with PD98059 (50 μM), SP600125 (10 μM) or SB203580 (20 μM) resulted in reduced expression of AP-1 and p-AP-1. However, the promoter activity and protein expression of CCR7 were significantly reduced in the PD98059- and SP600125-treated groups (compared to control and the TNF-α-treated groups, *P* < 0.05) but not in the SB203580-treated group. Therefore, ERK1/2 and JNK are involved in the TNF-α/AP-1/CCR7 signalling pathway.
  Taken together, these results indicated that TNF-α promotes AP-1 binding to the CCR7 promoter and upregulates CCR7 expression through the “ERK1/2-AP-1 and JNK-AP-1” pathways.
5. **Knock-down of CCR7 expression in NOZ cells using RNAi.**
  To find an effective siRNA to silence CCR7 expression, we designed three siRNA target sequences (si1, si2 and si3) from the human CCR7 gene (GenBank accession NO. NM_001301714.1) and a non-targeting control (siNC) (Fig. 4a). CCR7 protein in NOZ cells transiently transfected with siRNA oligos was detected by Western blotting. The results showed that the si3 oligos effectively knocked down the expression of CCR7 in NOZ cells compared to the negative control and siNC groups (Fig. 4b, c). Next, we used a lentiviral vector expressing the si3/shRNA construct (named LV-shCCR7) and a control vector containing a non-targeting sequence (named LV-shNC) to establish stably expressing cell lines. As shown in Fig. 4d, e, the protein level of CCR7 in the LV-shCCR7 group (NOZ cells stably infected with lentiviral CCR7 shRNA) was significantly decreased relative to the NOZ or LV-shNC (NOZ cells infected with empty vector) group (*P < 0.05). These cells were then used for the following cell and animal experiments.

**Fig. 4:**
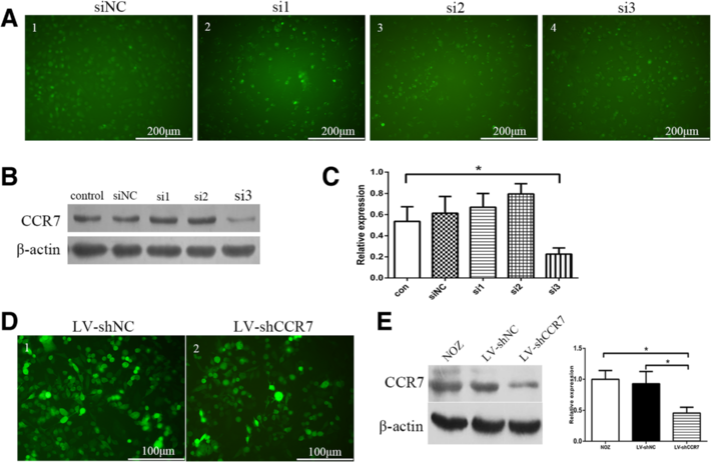
Expression of CCR7 in NOZ cells using RNAi technology. **a** Three siRNA oligos targeting the CCR7 gene (si1, si2 and si3) and a non-targeting control (siNC) were designed and chemically synthesized, and then transiently transfected into the NOZ cells. **b**, **c** The CCR7 protein in NOZ cells transfected with siRNA oligos was detected by Western blotting. The relative expression of CCR7 in the cells transfected with si3 oligos was significantly reduced compared to the negative control and siNC groups (*P < 0.05). **d** Construction of a NOZ cell line stably expressing lentiviral CCR7 (producing si3 sequence) or NC shRNA and a green fluorescent protein sequence. The cells were observed under a fluorescence microscope with blue light. **e** The CCR7 expression of NOZ cells stably transfected with LV-shCCR7 and LV-shNC was analyzed by western blotting. The protein level of CCR7 in the LV-shCCR7 group was significantly decreased relative to the LV-shNC group (*P < 0.05)
6. **TNF-α promotes GBC cells migrating to LECs through upregulation of CCR7.**
  To determine the role of CCR7 in the directional migration of GBC cells to LECs and whether TNF-α enhanced this migration and invasiveness, a transwell assay was performed. Following staining with H&E, 5 different fields (×400, magnification) were counted to determine the numbers of migrated and invaded cells. Figure 5a, b shows that the total number of cells in the NOZ + LEC group that migrated and invaded through the transwell polycarbonate filter was significantly higher than that of the cells in the NOZ group (*P* < 0.05), suggesting that NOZ cells directionally migrated to HDLECs. It was demonstrated that TNF-α dramatically enhanced the migratory ability of NOZ cells to HDLECs (Fig. 5b, c, NOZ + LEC + TNF group vs NOZ + LEC group, *P* < 0.05), and this effect was impaired when the CCR7 was knocked down by lentiviral-mediated shRNA (Fig. 5c–e, LV-shCCR7 + LEC + TNF group vs NOZ + LEC + TNF/LV-shNC + LEC + TNF groups, *P* < 0.05).

**Fig. 5:**
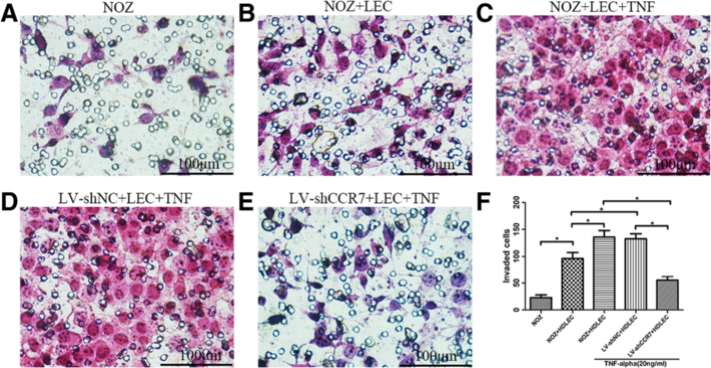
Effect of the TNF-α-CCR7 axis on the directional migration of GBC cells. **a** Migratory ability of the group that contained NOZ cells in the upper chamber only, without HDLECs in the lower chamber. **b** NOZ cells in the upper chamber and HDLECs in the lower chamber. **c** NOZ cells treated with 20 ng/mL TNF-α in the upper chamber and HDLECs in the lower chamber. **d** LV-shNC cells treated with 20 ng/mL TNF-α in the upper chamber and HDLECs in the lower chamber. **e** LV-shCCR7 cells treated with 20 ng/mL TNF-α in the upper chamber and HDLECs in the lower chamber. **f** Numbers of migrated cells in all 5 groups (*P < 0.05)
7. **TNF-α promotes lymph node metastasis through the upregulation of CCR7 in the orthotopic transplantation of GBC.**
  To observe whether TNF-α promotes lymph node metastasis through upregulation of CCR7 in vivo, we established two orthotropic xenograft models by planting NOZ and LV-shCCR7 cells in the gallbladder of nude mice (Fig. 6a). After 2 weeks, TNF-α or normal saline was injected into the peritoneal cavity of nude mice three times a week for three weeks. Lymph node metastases (LNM) of GBC were observed with the naked eye (Fig. 6c) and confirmed by HE staining (Fig. 6d). As shown in Table 5, TNF-α increased the LNM of orthotropic xenograft tumours compared to the control group, and this effect was impaired when the CCR7 was knocked down by lentiviral-mediated shRNA (LV-shCCR7 group). We also observed ascites and hepatic metastases in some mice (Fig. 6b).

**Fig. 6:**
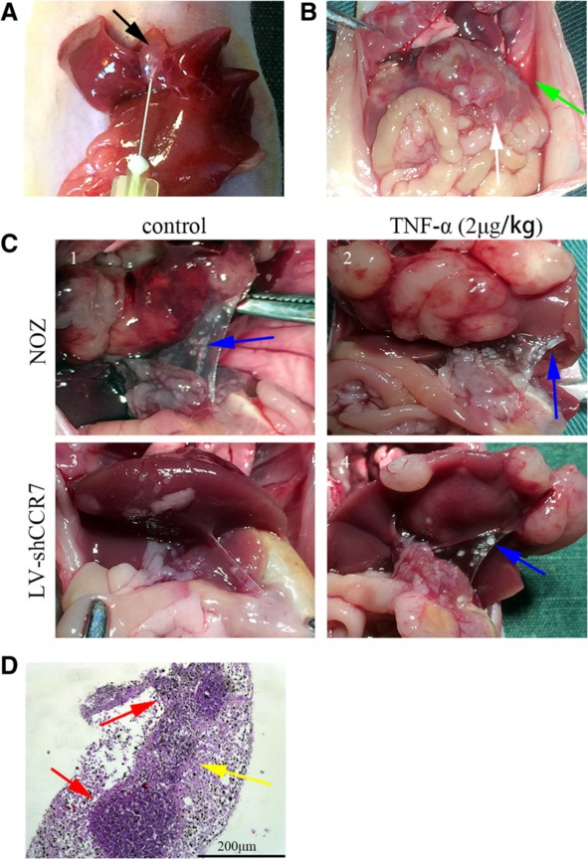
The TNF-α-CCR7 axis is involved in lymph node metastasis of GBC in vivo. **a** Establishment of orthotopic xenograft models of GBC in nude mice. After anaesthesia and exposure of the gallbladder (*black arrow*), one of two NOZ cell lines (NOZ or LV-shCCR7) was injected into the cavity of the gallbladder. **b**-**c** Gross anatomy of the nude mice. Ascites (*green arrow*) and hepatic metastasis (*white arrow*) were observed in the orthotopic xenograft models (**b**). The metastatic lymph nodes (*blue arrow*) are mainly located in the hepatoduodenal ligament (**c**). **d** Lymph node metastasis was further confirmed by H-E staining (200×). The invasive tumour cells (*yellow arrow*) could be observed in the lymphoid follicles (*red arrow*)

**Table 5:** Lymph node metastasis (LNM) of the orthotopic xenograft tumours in nude mice

	control	TNF-α(2 μg/kg)
NOZ	7/12	12/12*
LV-shCCR7	2/12*	4/12

*P < 0.05

## Discussion

Chemokine receptors and ligands play a critical role in the control of directional migration of many cell types, such as inflammatory cells [23, 24], immune cells and lymphocytes [12, 25]. Similar to the process of inflammatory cell recruitment and lymphocyte homing, chemokine receptors and ligands play important roles in mediating tumour cell transfer to target organs. Recently, many studies demonstrated that chemokine receptors and ligands are involved in tumourigenesis and tumour development. As mentioned above, CC chemokine receptor 7 (CCR7) is associated with lymphatic metastasis in certain types of tumours. However, the relationship between CCR7 and the lymph node metastasis of gallbladder cancer has not yet been reported.

In the present study, we used immunohistochemical techniques to investigate the expression of CCR7 in 42 gallbladder cancer specimens and 22 samples of normal gallbladder tissues adjacent to the cancer site. The results indicated that CCR7 is overexpressed in gallbladder cancer tissues, and is at low expression in normal tissues. The correlation with clinicopathologic factors was also analyzed. The high expression of CCR7 was significantly correlated with the AJCC staging and lymph node metastasis of GBC patients, which suggests that CCR7 may promote the development, especially the lymphatic metastasis, of GBC. Moreover, CCR7 expression was confirmed in all three GBC cell lines: NOZ, GBC-SD, and SGC-996. Against these backgrounds, we speculated that high expression of CCR7 may promote lymph node metastasis in GBC.

TNF-α is the key player in cancer-related inflammation, which can promote carcinogenesis, tumour growth, invasion, angiogenesis and epidermal-mesenchymal transition (EMT) [26–29]. However, little is known about the relation between TNF-α and lymphatic metastasis of the malignancies. In our previous study, we found that TNF-α can promote lymphangiogenesis and lymph node metastasis of GBC through upregulation of vascular endothelial growth factor C (VEGF-C) [8]. However, factors that are involved in the spread of GBC cells to the lymphatic system induced by TNF-α have been less well studied. Thus, we speculated that TNF-α might promote lymphatic metastasis of gallbladder cancer through upregulation of CCR7. We first analyzed the relationship between TNF-α and CCR7 via the detection of 20 clinical samples, and found that the level of TNF-α in the bile of GBC patients with strong CCR7 staining was significantly higher than that of patients with negative/week/moderate staining, which suggests that TNF-α is positively correlated with CCR7. Next, we employed exogenous TNF-α to stimulate the NOZ and SGC-996 cell lines, and found that TNF-α can significantly increase the expression of CCR7 in a dose- and time-dependent manner. These meaningful results prompted us to further explore the specific molecular mechanism underlying the TNF-α induced upregulation of CCR7.

TNF-α has dual effects on tumours (anti-cancer or pro-cancer effect), which are due to the activation of different downstream signalling pathways followed by the combination of TNF-α and its receptor TNFR1 [30]. According to the literature, three signalling pathways associated with TNF-α-induced tumour development are described below: the TNF-α - TNFR1 - signalling complex - MAP3K3/MEKK3/TAK1 - NF-κB pathway, the TNF-α - TNFR1 - signalling complex - MAP3K (ASK1) - JNK or p38 MAPK - AP-1 pathway and the TNF-α - TNFR1 - Ras - Raf - MEK1 - ERK1/2 - AP-1 pathway [31]. Thus, we first analyzed the activity of the CCR7 promoter (Fig. 3a) and searched for potential binding sites of NF-κB or AP-1 in the -243 nt to -108 nt region of the CCR7 promoter by using the TFbind and Promoter Scan programs. Two putative AP-1 binding sites were found in this region, whereas NF-κB sites were not identified (Fig. 3b). Site-directed mutagenesis and ChIP assays were then used to reveal that the AP-1(2) site is crucial for the activity of CCR7 promoter but not the AP-1(1) binding site. To verify the molecules upstream of CCR7, we used siRNA to knock down AP-1. The promoter activity and the protein level of CCR7 were consequently decreased in both groups, with or without TNF-α treatment. Subsequently, we analyzed the effect of inhibition of MAPK pathway members on the promoter activity and protein expression of CCR7. The results suggested that ERK1/2 and JNK are upstream of AP-1. These data confirm the existence of the “TNF-α - ERK1/2 - AP-1 - CCR7” and “TNF-α - JNK - AP-1 - CCR7” pathways in NOZ cells. Mburu YK et al. reported that NF-κB and AP-1 co-regulate CCR7 expression in metastatic squamous cell carcinoma of the head and neck [32]. The AP-1 site they found is consistent with our data. In another study, Côté SC et al. found that the transcription factors CREB-1, C/EBPα and C/EBPβ can bind the CCR7 promoter region in human monocytes [33]. These studies showed that multiple transcription factors are involved in the regulation of CCR7 expression, while the specific mechanism may differ in different cell types.

Next, we wanted to determine the role of the TNF-α - CCR7 axis in the migration of GBC cells to the lymphatic system. We first successfully established a NOZ cell line stably expressing lentiviral CCR7 shRNA which effectively silenced the expression of CCR7 (Fig. 4). Next, a transwell assay was performed. We seeded NOZ cells in the upper chamber of the transwell system and HDLECs in the lower chamber. The results indicated that NOZ cells directionally migrated to HDLECs, and TNF-α dramatically enhanced this migration. This enhancement induced by TNF-α was impaired when CCR7 was knocked down by lentiviral-mediated shRNA (Fig. 5). It was demonstrated that the lymphatic endothelial cells (LECs) secreted the chemokines CCL21 and CCL19, which are not secreted by blood endothelial cells (BECs) [34]. CCL21 and CCL19 are specific ligands for CCR7. Together with our data, we consider that the combination of CCL21/CCL19 (secreted by LECs) and CCR7 (located on the GBC cell membrane) prompts GBC cells to directionally migrate to the lymphatic vessels, eventually leading to lymph node metastasis.

To confirm our above conclusion in vivo, we conducted animal experiments. The results indicated that TNF-α can increase the lymph node metastasis of orthotopic xenograft tumours in nude mice, and this effect was impaired when CCR7 was silenced. These data demonstrate that CCR7 can mediate the TNF-α-induced lymphatic metastasis of gallbladder cancer in vivo. Consistent with our results, previous reports support the conclusion that CCR7 could promote lymph node metastasis. Emmett MS et al. observed that CCR7 mediates directed growth of melanomas towards lymphatics; they called this process in-transit metastasis [17]. Heather D. Cunningham et al. indicated that CCR7 mediates metastasis of breast cancer to the lymph nodes in mice [35].

## Conclusions

Our study suggests that CCR7 is highly expressed in GBC, and it mediates the TNF-α-induced lymphatic metastasis of GBC through the “ERK1/2 - AP-1 - CCR7” and “JNK - AP-1 - CCR7” pathways. These data indicate that CCR7 may be used as a potential treatment target in preventing lymphatic metastasis of gallbladder cancer.

## Abbreviations

AP-1: activator protein-1
CCR7: CC-chemokine receptor 7
GBC: gallbladder cancer
HDLECs: human dermal lymphatic endothelial cells
LNM: lymph node metastasis
TNF-α: tumour necrosis factor-alpha
